# Heinemann Medical Dictionary

**Published:** 1987-08

**Authors:** J. D. Davies


### Book Reviews

**HEINEMANN MEDICAL DICTIONARY**
**B. Lennox and M. E. Lennox**
**Wm. Heinemann Medical Books, London**
**ISBN 0 433 19154 6. Price £9.95**
**pp. 611+vi, paperback, 1986**

I confess to have been looking forward to reading this book for several months. In the end a review copy from the publishers has not been a disappointment.

The truth is that most dictionaries are very boring to their readers. The medical ones are usually the worst, even if they are designed to help our badly paid secretaries in their early months of a culture-shock state. Further, most such medical dictionaries are also impressively bound, North American and very expensive.

This medical dictionary from Messrs Heinemann is nothing of the kind. It appears in a humble paperback version, as well as in hardback. Don't be deceived by the deceptively conventional blue-grey cover of the paperback. It is dynamite.

The senior author is none other than Bernard Lennox. All pathologists, young and old, will know Professor Lennox' love of linguistic peculiarity. More general

*(continued on page 86)*

Book Review *(continued from page 81)*
medical readers may have already, if unwittingly, benefitted from his rôle as a consultant to the recent supplementary additions to the mighty *Oxford English Dictionary.*

The declared aim of this substantial, and yet more practical book is to help all sorts of medical practitioners understand each other. I think that it does much more than that. By calling our curious language into question, it also helps us to understand ourselves.

For instance, how often have you written De Morgan, Demodex or diarrhoea (the English form of which is not included) without having thought about their original source? Did you know that Langhans and Langerhans (of the giant cell and pancreas) were not the same chap, and yet were born within eight years of one another? Is a French chignon just a further excuse to cross the Channel for yet another conference? Or, what sort of fashionable cancer-related organic compound is putrescine? What on earth does menoxenia mean? Did you realise that we unconsciously echo a 13th century medical theory in talking about gout?

Obviously there are caveats. Personally I think the Lennoxian *fetal* is objectionably precious and quite alien to current spoken English. In the same way Professor Lennox clearly finds our own traditional English *foetus*— of 15th century precedence, also earnestly used by the gas-law William Boyle, FRS, in 1660 — and the corresponding adjective *foetal* anachronistic (see, *inter alia,* for contrary anti-Lennoxian views the OED itself in F–G, vol. 4, p. 379, 1933). Essentially this heated debate concerns the modern typographical reproduction of the long-lost written diphthongs of old English. To my mind there is no doubt that the Southern English pronunciation of foetus involves an initial diphthongal vowel. There is clearly much to debate in this area. As you will understand not everyone agrees on these issues. This is not to berate Professor Lennox; I admire his good intention. He also helpfully highlights the recent if odd English use of *homoeobox,* still loyally used by *Nature,* even though part of its editorial office has moved to the United States. I trust that everyone interested will express their own view both in this journal and elsewhere. A consensus may help to change, or to stabilise the form of our medico-scientific English language.

Beyond this, there are deficits which are obviously not to be laid at the door of these two accomplished dictionarists. However, for example the meaning and etymology of *Infarct* do not appear. Any student or postgraduate examiner will know that the '99% of doctors' mentioned in the Preface do not have a clear concept of the term. For this reason alone it needs inclusion in this dictionary.

None the less we can all share wholeheartedly in the impressive scholarship of this exciting and novel dictionary. A generous 20,000 verbal entries are included in the 600 pages of the book. It is fascinating, addictive and instructive. The Lennoxes have surely managed to find many new words which are novel even in your own field. I should like to congratulate them on 'ELP', meaning Elastase-like-protease which I freely admit was new to me. You too, may be equally surprised about their finds of other words peculiar to your own speciality.

Don't wait for the likely second edition of this splendid new medical dictionary: take the opportunity now. By the time that my own copy of the book has lost its binding and the odd pages, rest assured I'll also be there in the local bookshop placing an order for a replacement. Better still, why not take up the publishers' offer of a free copy of a second edition in return for suggested corrections or additions to this first edition? We can all gain from your personal contributions. Words are not private property. They need sharing, debate and a decent airing. The wait for this new medical dictionary has been well worthwhile. I am sure that you will agree.

J. D. Davies

